# Single-Cell Transcriptomics Reveals the Molecular Anatomy of Sheep Hair Follicle Heterogeneity and Wool Curvature

**DOI:** 10.3389/fcell.2021.800157

**Published:** 2021-12-21

**Authors:** Shanhe Wang, Tianyi Wu, Jingyi Sun, Yue Li, Zehu Yuan, Wei Sun

**Affiliations:** ^1^ College of Animal Science and Technology, Yangzhou University, Yangzhou, China; ^2^ College of Veterinary Medicine, Yangzhou University, Yangzhou, China; ^3^ Joint International Research Laboratory of Agriculture and Agri-Product Safety of Ministry of Education of China, Yangzhou University, Yangzhou, China

**Keywords:** single-cell transcriptome, sheep, hair follicle, cellular heterogeneity, wool curvature

## Abstract

Wool is the critical textile raw material which is produced by the hair follicle of sheep. Therefore, it has important implications to investigate the molecular mechanism governing hair follicle development. Due to high cellular heterogeneity as well as the insufficient cellular, molecular, and spatial characterization of hair follicles on sheep, the molecular mechanisms involved in hair follicle development and wool curvature of sheep remains largely unknown. Single-cell RNA sequencing (scRNA-seq) technologies have made it possible to comprehensively dissect the cellular composition of complex skin tissues and unveil the differentiation and spatial signatures of epidermal and hair follicle development. However, such studies are lacking so far in sheep. Here, single-cell suspensions from the curly wool and straight wool lambskins were prepared for unbiased scRNA-seq. Based on UAMP dimension reduction analysis, we identified 19 distinct cell populations from 15,830 single-cell transcriptomes and characterized their cellular identity according to specific gene expression profiles. Furthermore, novel marker gene was applied in identifying dermal papilla cells isolated *in vitro*. By using pseudotime ordering analysis, we constructed the matrix cell lineage differentiation trajectory and revealed the dynamic gene expression profiles of matrix progenitors' commitment to the hair shaft and inner root sheath (IRS) cells. Meanwhile, intercellular communication between mesenchymal and epithelial cells was inferred based on CellChat and the prior knowledge of ligand–receptor pairs. As a result, strong intercellular communication and associated signaling pathways were revealed. Besides, to clarify the molecular mechanism of wool curvature, differentially expressed genes in specific cells between straight wool and curly wool were identified and analyzed. Our findings here provided an unbiased and systematic view of the molecular anatomy of sheep hair follicle comprising 19 clusters; revealed the differentiation, spatial signatures, and intercellular communication underlying sheep hair follicle development; and at the same time revealed the potential molecular mechanism of wool curvature, which will give important new insights into the biology of the sheep hair follicle and has implications for sheep breeding.

## Introduction

Wool is the critical textile raw material which is produced by the hair follicle of sheep. Therefore, it has important implications to investigate the molecular mechanism driving hair follicle development (Clavel et al., 2012; Liu et al., 2014). However, due to high cellular heterogeneity and the insufficient cellular, molecular, and spatial characterization of the hair follicle in sheep (Adelson et al., 2004; Yu et al., 2011; Saxena et al., 2019), the molecular mechanisms involved in the hair follicle development of sheep remains largely unknown.

The hair follicle is a unique composite organ, consisting of a variety of epithelial and mesenchymal lineage cells in a sophisticated structure (Rice and Rompolas, 2020; Zou et al., 2021). It cycles through phases of anagen, catagen, and telogen, which rely on tightly coordinated mesenchymal–epithelial interactions (Rendl et al., 2005; Avigad Laron et al., 2018). Hair follicle stem cell (HFSC) and dermal papilla cell (DPC) play a crucial role in hair follicle cycles, which was demonstrated by hair regeneration assay *in vitro* using the above cells (Greco et al., 2009; Kageyama et al., 2018; Lee et al., 2020). The research of hair follicles progressed rapidly since the molecular markers of HFSC and DPC had been identified (Jaks et al., 2008; Ohyama et al., 2012; Rompolas and Greco, 2014), such as keratin 15 (K15), CD34 in mice, and CD200 in humans for HFSC as well as SOX2 for DPC (Jaks et al., 2010; Rompolas et al., 2012; Morgan, 2014; Molina et al., 2018), which allowed isolation and characterization of these cells from follicles using fluorescence-activated cell sorting (FACS) (Rendl et al., 2005; Rezza et al., 2016). Besides, recent studies showed that immune cells, adipose cells, etc. in the skin play critical roles in regulating hair follicle development (Hsu et al., 2014). However, due to the lack of bulge structure and few available antibodies for sheep (Rogers, 2006), the marker genes of HFSC and DPC as well as other cells of the hair follicle in sheep are still elusive. Therefore, identification of the molecular markers and the signatures of all kinds of cells in hair follicles contribute to understanding the intercellular interaction and the molecular mechanism underlying hair follicle development in sheep.

Wool has curly characteristics in general, which is critical for wool performance and wool quality. It is still unclear on the genetic mechanism of wool curvature (Harland et al., 2018; Xiao et al., 2019). Through transgenic mice models and naturally mutated mice, the genes affecting hair curvature have been revealed (Johnson et al., 2003; Nakamura et al., 2013). Besides, a few genetic variations and genes, such as *KRT71*, *KRT74*, *TCHH*, *CUTC*, *IGFBP5*, and *WNT10A*, have been identified using genome-wide association study (GWAS) and whole-genome sequencing (WGS) (Cadieu et al., 2009; Harel and Christiano, 2012; Demars et al., 2017; Liu et al., 2018). Meanwhile, Eda and TGFβ signaling have been demonstrated to play an important role in regulating hair curvature (Fujimoto et al., 2008; Nissimov and Das Chaudhuri, 2014). Thibaut revealed that hair curliness was programmed from the bulb and was associated with asymmetry in differentiation programs (Thibaut et al., 2005). Furthermore, Driskell demonstrated a previously unrecognized heterogeneity in DPCs and revealed that Sox2-positive DPCs specified particular hair follicle types in mice, in which Sox2 was only expressed in the DPC of guard/awl/auchene follicles rather than in zigzag follicles (Driskell et al., 2009). Hu sheep, our research object, is famous for lambskin and high reproductive traits. Interestingly, Hu sheep could be segregated into two populations including curly population and straight population in terms of wool characteristics, which provide an excellent research material to study the biology of wool curvature. However, the mechanism of wool curvature is still unknown because of the inaccessibility in isolation and characterization of specific cells precisely.

Powered by single-cell RNA sequencing (scRNA-seq), the above problems could be well solved now. Single-cell gene expression studies enable one to profile transcriptional regulation in highly heterogeneous cell populations and complex biological processes (Chovatiya et al., 2021), which was applied in hair follicle biology researches (Joost et al., 2016; Ge et al., 2020; Haensel et al., 2020; Takahashi et al., 2020; Ge et al., 2021). Recently, Joost et al. used scRNA-seq to reveal unprecedented molecular details of cell types and states coordinating hair growth in mice (Joost et al., 2020). Nevertheless, such studies are lacking so far in sheep. From this perspective, we used 10×Genomics scRNA-seq to systematically dissect the cellular heterogeneity and to reveal the molecular pathways underlying hair follicle development and wool curvature using Hu sheep composed curly population and straight population. We provided a systematic view of the transcriptional landscape of sheep hair follicles and revealed the differentiation and spatial signatures underlying sheep hair follicle heterogeneity and wool curvature.

## Materials and Methods

### Animals

Hu sheep from Suzhou sheep breeding farm (Suzhou, Jiangsu, China) were used in this study. According to the wool length and curvature, curly wool lambs and straight wool lambs were selected to obtain lambskin samples. After infiltration anesthesia through hypodermic injection of 2% lidocaine hydrochloride (10 mg), skin samples approximately 1 cm^2^ and 2 mm deep were harvested from the body side of lambs. Subsequently, the wound was sewed up, and the sample was divided into two parts; one was kept in Dulbecco's modified essential medium (DMEM)/F12 medium (Gibco™, Cat#31331093) with penicillin-streptomycin (Gibco™, Cat#15240062), and the other was fixed with 4% paraformaldehyde (Solarbio, Cat#P1110) for subsequent analysis. All the experimental procedures with the sheep used in this study received prior approval from the Experimental Animal Manage Committee of Yangzhou University.

### Preparation of Single-Cell Suspension

To dissociate lambskin into single cells, skins from curly wool individuals (*n* = 3) and straight wool individuals (*n* = 3) were pooled from each group. In order to ensure the quantity of hair follicle cells for each group, we adopt two strategies for dissociation. For strategy 1, single hair follicles were isolated and dissociated into single cells. For strategy 2, the whole skin was dissociated into single cells. Finally, single cells from the two strategies were mixed as the final example. For hair follicle dissociation, skins were cut into small pieces, and the hair follicles were isolated via microdissection using a stereoscope. Collected hair follicles were incubated with pre-warmed (37°C) TrypLE (Gibco™, Cat#12604013) for a total of 15 min. Then, 3% bovine serum albumin (BSA) solution (Sigma-Aldrich, Cat#A1933) was added to the dissociated cell suspension to inactivate TrypLE enzymatic activity. For skin tissues, 2 mg/ml collagenase IV (Sigma-Aldrich, Cat#C4-BIOC) was used to digest lambskin tissues at 37°C for a total of 30 min. Then, the samples were centrifuged at 1,000 rpm for 5 min, and the supernatant was discarded. The precipitate was incubated with pre-warmed TrypLE (Gibco™, Cat#12604013) for a total of 15 min. After that, the skin tissues were mechanically dissociated into single-cell suspension through pipetting. The cell suspensions were then filtered through a 40 μm nylon cell strainer (Sangon Biotech, Cat#12604013). Subsequently, the suspension from hair follicles and skins were mixed and resuspended in 3% BSA after 3 times washing with cold 3% BSA solution. Viability and live cell count of samples were determined using Trypan blue and Countess II. The final cell concentration was around 1,000 cells/μl, with a cell viability greater than 90%.

### scRNA-Seq cDNA Library Preparation and Sequencing

Single-cell barcoding and library preparation were performed based on the 10×Genomics scRNA-seq platform (10×Genomics, Pleasanton, CA, United States) as described (Ge et al., 2020). Approximately 8,000 cells per sample were added to a single-cell master mix and to barcode with 10×barcoded gel beads. 10×Barcoded cDNA libraries were constructed using a 10×Genomics Chromium barcoding system following the manufacturer's instructions. Illumina HiSeq X Ten sequencer (Illumina, San Diego, CA, United States) was used for sequencing, and pair-ended 150 bp (PE150) reads were generated for downstream analysis.

### scRNA-Seq Data Analysis

The scRNA-seq data analysis was performed as previously reported (Ge et al., 2020). Briefly, The CellRanger (v2.2.0) software was used for analyzing raw sequencing data according to the 10×Genomics official pipeline (https://support.10xgenomics.com/single-cell-gene-expression/software/pipelines/latest/what-is-cell-ranger). The reads were aligned to sheep reference genome (https://ftp.ncbi.nlm.nih.gov/genomes/refseq/vertebrate_mammalian/Ovis_aries/latest_assembly_versions/GCF_002742125.1_Oar_rambouillet_v1.0/) using wrapped STAR software (https://github.com/alexdobin/STAR).

The quality control (QC) and cell clustering were analyzed with single-cell RNA seq Seurat software (v2.3.4) based on R environment (R version: 3.6.3, https://www.r-project.org/) following the online guide (https://satijalab.org/seurat/). We performed a clustering analysis on the integrated dataset based on UMAP (Uniform Manifold Approximation and Projection for Dimension Reduction) algorithm implemented in Seurat.

To construct single-cell pseudotime differentiation trajectory, we used Monocle (v 2.10.0) to order single cells along pseudotime according to the official tutorial (http://cole-trapnell-lab.github.io/monocle-release/docs/#constructing-single-cell-trajectories). Metascape (http://metascape.org/gp/index.html#/main/step1) was used to perform gene ontology (GO) analysis to investigate gene functions in each gene cluster.

### Identify Differentially Expressed Genes Between Curly Wool Lambskin Group and Straight Wool Lambskin Group in Specific Cells

We performed differentially expressed genes (DEGs) analysis to look at the difference between different groups in specific cell cluster using Seurat software following the official tutorial (https://satijalab.org/seurat/v3.0/immune_alignment.html). Here, we used the “avg.Idents” function to take the average expression of specific cells between different groups and used the “genes.to.label” function to generate the scatter plots, highlighting genes that exhibit dramatic changes. We used the “FindMarkers” function to identify the DEGs between the curly wool lambskin group and straight wool lambskin group in specific cells.

### Cell to Cell Ligand–Receptor Interaction Analysis

CellChat (http://www.cellchat.org/) (Jin et al., 2021) was used to analyze the intercellular communication networks from scRNA-seq data. We performed analysis according to R toolkit CellChat (https://htmlpreview.github.io/?https://github.com/sqjin/CellChat/blob/master/tutorial/CellChat-vignette.html). To infer the specific ligand–receptor pairs, we compared cell type-specific DEGs identified by Seurat with annotated ligand–receptor pairs compiled by Skelly (Skelly et al., 2018) as described previously (Ge et al., 2020).

### DPCs Isolation and Culture

Skin tissues were cut into small pieces with scissors, and single hair follicles were isolated using ophthalmic forceps (Sigma-Aldrich, Cat#F3767-1EA). Next, single hair follicles were incubated with TrypLE (Gibco™, Cat#12604013) at 37°C for 15 min, then the hair bulb was mechanically cut down with a 1 ml syringe needle, and the compact dermal papilla (DP) was squeezed out from the hair bulb. The obtained cells were collected and cultured using DPC medium in adherent culture dishes. The DPC medium consisted of DMEM/F12 media (Gibco™, Cat#31331093) supplemented with 20% fetal bovine serum (FBS, Gibc™, Cat#16140071) 40 ng/ml recombinant human fibroblast growth factor (FGF)-basic (bFGF, PeproTech, Cat#100-18B), penicillin (100 U/ml), and streptomycin (100 mg/ml). DPCs migrated from compact DP at 3–4 days after seeding. After culturing 10 days, we passaged DPCs. After that, cells were passaged every 2 days. DPCs in passage 2 were used to conduct immunofluorescence staining.

### Immunofluorescence Staining

For skin tissue section samples and cell samples, we performed immunofluorescence staining as described (Ge et al., 2020; Zhang et al., 2021). The primary antibodies and secondary antibodies used in this study are listed in Table 1. Hoechst 33,342 (Beyotime Biotechnology, Cat#C1022) was used for nuclei staining, and the slides were finally mounted with Anti-Fade Fluorescence Mounting Medium—Aqueous (Abcam, Cat#ab104135). Fluorescent pictures were taken under a LEICA TCS SP5 II confocal microscopy (Leica Microsystems GmbH, Wetzlar, Germany).

**TABLE 1:** List of antibodies used for immunofluorescence staining.

Antibody	Manufacture	Cat no
VIM antibody	Santa Cruz	sc-6260
IGF1 antibody	Santa Cruz	sc-518040
IGFBP3 antibody	Santa Cruz	sc-374365
KRT71 antibody	GeneTex	GTX107343
CUX1 polyclonal antibody	proteintech	11733-1-AP
Anti-BMP2 antibody	Abcam	ab214821
Anti-Corin antibody	Abcam	ab255812
VDR monoclonal antibody	proteintech	67192-1-Ig
Anti-PCNA antibody	Abcam	ab29
Anti-ACTA1 rabbit polyclonal antibody	Sangon Biotech	D121592
β-Catenin rabbit polyclonal antibody	Beyotime	AF5126
CYTB polyclonal antibody	proteintech	55090-1-AP
Anti-VCAN rabbit polyclonal antibody	Sangon Biotech	D223532
Goat anti-mouse IgG H&L (Alexa Fluor^®^ 555)	Abcam	Ab150114
Goat anti-rabbit IgG H&L (Alexa Fluor^®^ 555)	Abcam	Ab150078
Goat anti-rabbit IgG H&L (Alexa Fluor^®^ 488)	Abcam	Ab150077

## Results

### Single-Cell Sequencing and Characterization of Cellular Heterogeneity

**FIGURE 1:**
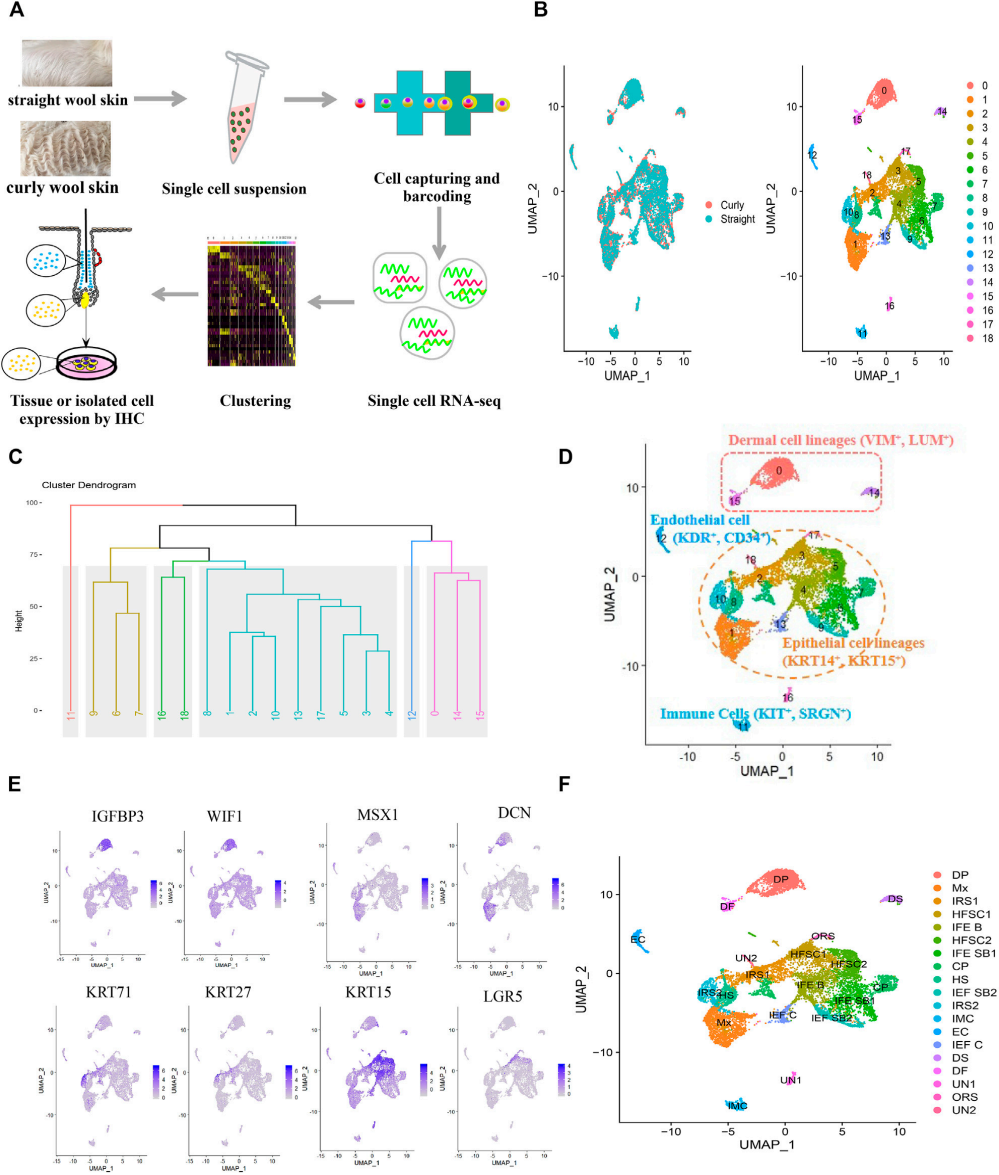
Single-cell sequencing and characterization of cellular heterogeneity of sheep lambskin. **(A)** Overview of the experimental workflow. **(B)** UMAP plot of sheep lambskin single skin cells. The left plot depicts UMAP plot of the integrated dataset from two different groups including straight wool and curly wool groups, and cells are different color-coded. The right plot depicts 19 transcriptional distinct cell clusters, and cells are color-coded with cluster information. Cells in the same cluster represent high similarity in transcriptome profile. **(C)** Hierarchical clustering on the 19 cell clusters. Six major branches with different color are revealed. **(D)** Characterization of major cell types in the sheep lambskin tissues in the UAMP plot. Cells are color-coded and are labeled with their cell identity. **(E)** Feature plot depicts representative gene expression of dermal cell, matrix, and HFSC. **(F)** Annotation of each cell cluster in the UAMP plot.

To gain insight into the transcriptional heterogeneity of the anagen hair follicle cells and to clarify the molecular mechanisms of wool curvature, we performed scRNA-seq on Hu sheep skins from straight wool individuals and curly wool individuals (Figure 1A). We detected 19,097 genes in skin cells for the straight wool group and 19,059 genes for the curly wool group. After QC, we obtained 15,830 single-cell transcriptome profiles from these two groups (7,263 for the straight wool group and 8,567 single cells for the curly wool group) (Supplementary Figure S1).

To dissect the cellular heterogeneity, we then performed UMAP dimension reduction analysis using all the single cells, in which the straight and curly datasets were integrated to identify differences and similarities in cellular composition. Unbiased clustering identified 19 clusters according to their gene expression profiles, and all cell clusters were present in straight and curly wool skins (Figure 1B), suggesting that differential gene expression profiles rather than cellular components played an important role in wool curvature. To further characterize these cell clusters, hierarchical clustering was performed on the 19 cell clusters. The result revealed six major branches (clusters 0, 14, and 15; clusters 16 and 18; cluster 11; clusters 1, 2, 3, 4, 5, 8, 10,13, and 17; clusters 6, 7, and 9; and cluster 12) (Figure 1C), which reflected the relationship among different clusters to some extent, since clusters in the same branch have closer relationship. Through analyzing the expression of a series of well-recognized cell marker genes, we manually annotated these clusters into five major cell groups: epithelial cell lineages (clusters 1, 2, 3, 4, 5, 6, 7, 8, 9, 10, 13, and 17) expressed specific keratins such as KRT14, KRT15, KRT1, and KRT71 (Schweizer et al., 2007); dermal cell lineages (clusters 0, 14, and 15) expressed VIM and LUM (Kong et al., 2006); immune cells (cluster 11) expressed SRGN and KIT (Ge et al., 2020; Joost et al., 2020); endothelial cells (cluster 12) expressed KDR and CD34 (Detmar et al., 1998; Ge et al., 2020); and unknown cells (clusters 16 and 18) (Figure 1D). Furthermore, epithelial cell lineages could be divided into hair follicle epithelial cell lineages and interfollicular epithelial cell lineages according to the differentially expressed keratins. It is noteworthy that the characterization of cell types was consistent with our hierarchical clustering analysis, which further confirmed our cell-type annotation.

To accurately identify each cell cluster, we compared the gene expression across the cell clusters based on differentiation expression analysis. Highly DEG expression profiles are shown in Supplementary Figure S2A to demonstrate cluster-specific expression. The full list of identified cell-type signature genes is documented in Supplementary Table S1. Then, we identified each cell cluster according to the expression of a series of well-recognized cell marker genes and cluster-specific genes. As a result, we revealed 14 major cell identities (Table 2): IGFBP3 and WIF1 highly expressing DPC cluster (cluster 0); MSX1 and DCN highly expressing matrix cluster (cluster 1); KRT71 and KRT27 highly expressing inner root sheath cell (IRS) clusters (cluster 2 and 10); KRT15 and LGR5 highly expressing HFSC clusters (clusters 3 and 5) (Figures 1E, F) etc., which showed the cellular heterogeneity of skin cells. In addition, we revealed some potential novel marker genes, such as CXCL14 specifically expressed in HFSC, ACTG2 specifically expressed in dermal sheath (DS) cell, and CRABP1and APOD specifically expressed in DPC (Supplementary Figure S2B). These specifically expressed genes should be further studied and validated.

**TABLE 2:** Cell types and their corresponding marker genes analyzed from scRNA-seq.

Cluster	Cell type	Markers	References
0	Dermal papilla (DP)	IGFBP3, SOX18, WIF1	Weger and Schlake (2005); Rendl et al. (2008); Driskell et al. (2009)
1	Matrix (Mx)	MSX1, DCN, ID3	Choi et al. (2011); Yang et al. (2017); Joost et al. (2020)
2, 10	Inner root sheath (IRS)	KRT71, KRT25	Schweizer et al. (2007); Moch et al. (2013)
3, 5	Hair follicle stem cells (HFSC)	KRT15, LGR5, CXCL14	Liu et al. (2003); Yang et al. (2017); Miyachi et al. (2018)
4	Interfollicular epidermis basal (IFE B)	POSTN, KRT14	Gu and Coulombe (2007); Arima et al. (2015)
6, 9	Interfollicular epidermis super-basal (IFE SB)	KRTDAP, KRT1, KRT2	Bazzi et al. (2007); Torma (2011)
7	Companion layer (CL)	KRT6A, KRT14	Gu and Coulombe (2007); Joost et al. (2020)
8	Hair shaft (HS)	KRT35, KRT84	Yu et al. (2011)
11	Immune cells (IMC)	FCER1G, SRGN, KIT	Ge et al. (2020); Joost et al. (2020)
12	Endothelial cells (EC)	CD34, KDR	Detmar et al. (1998); Ge et al. (2020)
13	Interfollicular epidermis basal, cycling (IFE C)	TOP2A, BIRC5, KRT5	Joost et al. (2020)
14	Dermal sheath (DS)	ACTA1, ACTA2	Paquet-Fifield et al. (2009)
15	Dermal fibroblasts (DF)	VIM, LUM	Gupta et al. (2019)
16, 18	Unknown (UN)	—	—
17	Outer root sheath (ORS)	VDR, FGF5	Ota et al. (2002); Joost et al. (2020)

**FIGURE 2:**
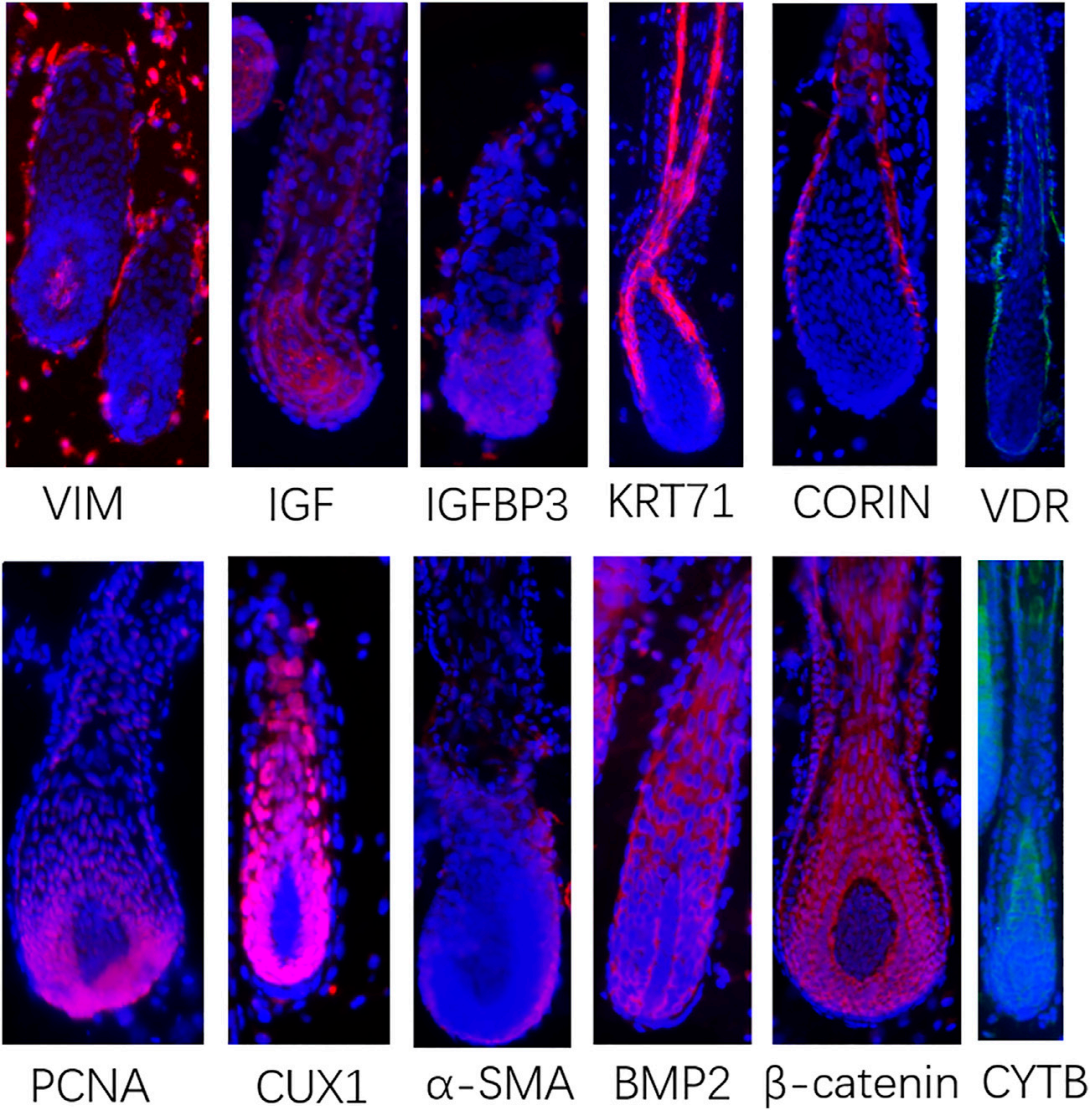
Immunofluorescence in skin tissue sections to verify the signature genes came from scRNA-seq. Red or green fluorescence indicates the expression pattern of target protein. Nucleus was stained with Hoechst in blue.

In order to verify the above-characterized cell types and these marker genes, we conducted immunofluorescence in skin tissue sections. The results were as follows: VIM were specifically expressed in dermal cell lineages, KRT71 were specifically expressed in IRS cells, VDR were specifically expressed in outer root sheath (ORS) cells, CORIN and α-SMA were specifically expressed in DS cells, PCNA were specifically expressed in matrix cells, IGF1 and IGFBP3 were highly expressed in DPCs, and CUX1 and CYTB were highly expressed in matrix cells and pre-cortex cells (Figure 2). In addition, we found that BMP2 and β-catenin, the most important signaling genes of the BMP and WNT pathways, were highly expressed in the anagen hair follicle epithelial cells. These results, together with cluster-specific marker gene expression indicated above, reinforced that the results of cell populations characterization are reliable. Together, these analyses here firstly characterized the different cell populations within the skin tissues of sheep.

### Application of Identified Marker Genes on DPCs Isolated *In Vitro*

**FIGURE 3:**
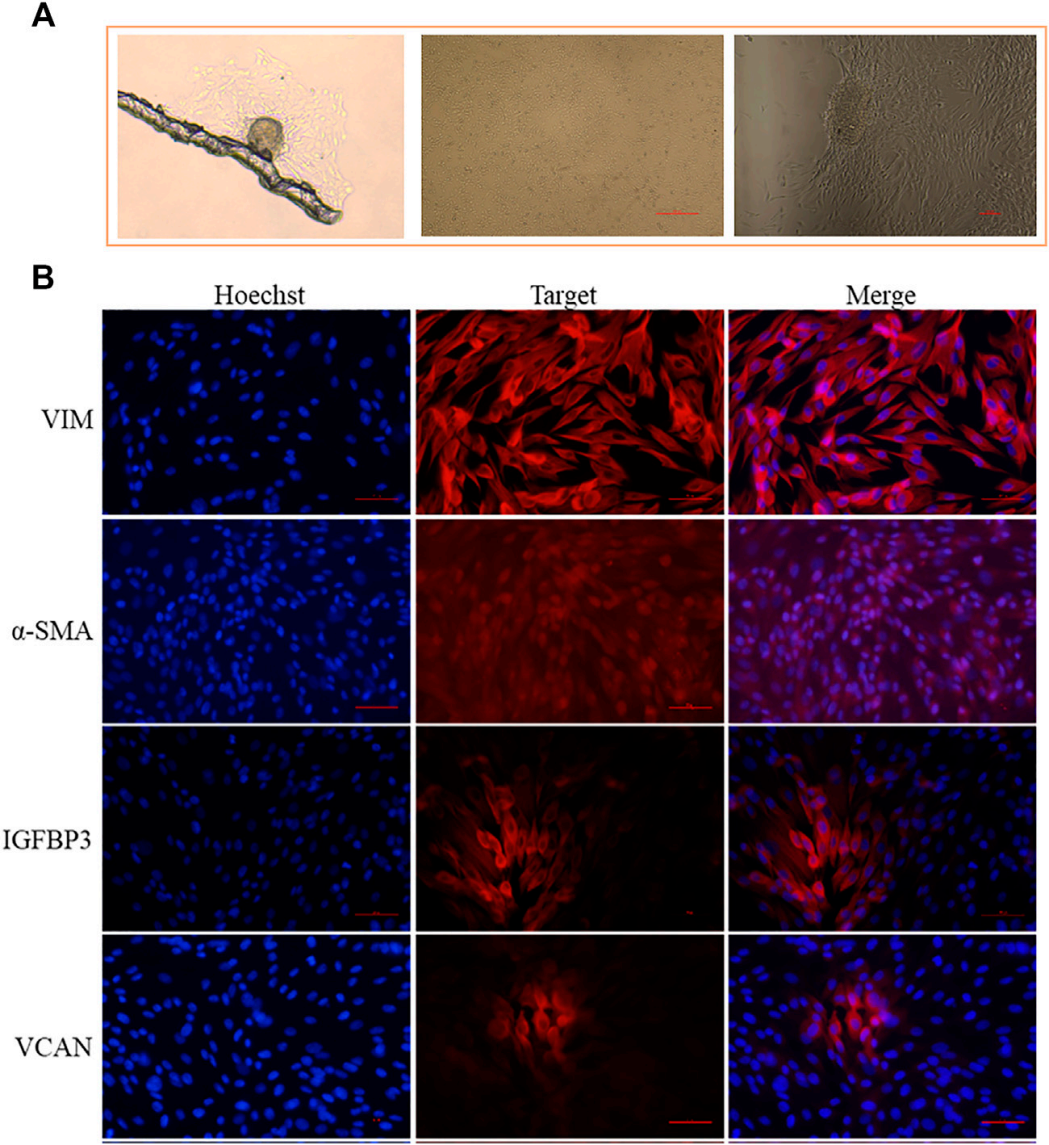
Application of identified marker genes on DPCs isolated *in vitro*. **(A)** Isolated DPCs exhibit a multilayer aggregative growth character. **(B)** Immunofluorescence analysis of VIM, α-SMA, IGFBP3, and VCAN expression in the isolated DPCs. Scale bars, 50 μm. Red fluorescence indicates the nuclear expression pattern of target protein. Nucleus was stained with Hoechst in blue.

Marker genes could be used for cell isolation and identification *in vitro*. Since we have successfully identified the marker genes of different cell populations based on scRNA-seq, we verified these marker genes on DPCs isolated *in vitro*. Through the mechanical and enzymatic isolation method, we successfully isolated the DPCs of Hu sheep, which showed a multilayer aggregative growth character *in vitro* culture (Figure 3A). Using well-recognized cell markers of DPCs (VCAN and α-SMA) and novel marker gene (*IGFBP3*) identified by scRNA-seq as well as marker gene of dermal lineage cells (*VIM*), we verified the DPCs in the primary culture through immunofluorescence. As a result, all the cells exhibited positive expression of VIM and α-SMA, further emphasizing their DPCs identity. Meanwhile, VCAN and IGFBP3 were highly expressed in cells with aggregative character (Figure 3B), which suggested VCAN and IGFBP3 were closely related with the aggregative growth character of DPCs. Since VCAN have been demonstrated to play a critical role in DPC aggregative growth (Yang et al., 2012), and IGFBP3 exhibited the same expression pattern with VCAN, we believed that IGFBP3 was closely related with the aggregative growth character of DPCs.

### Reconstruction of the Hair Follicle Epidermal Differentiation Process

**FIGURE 4:**
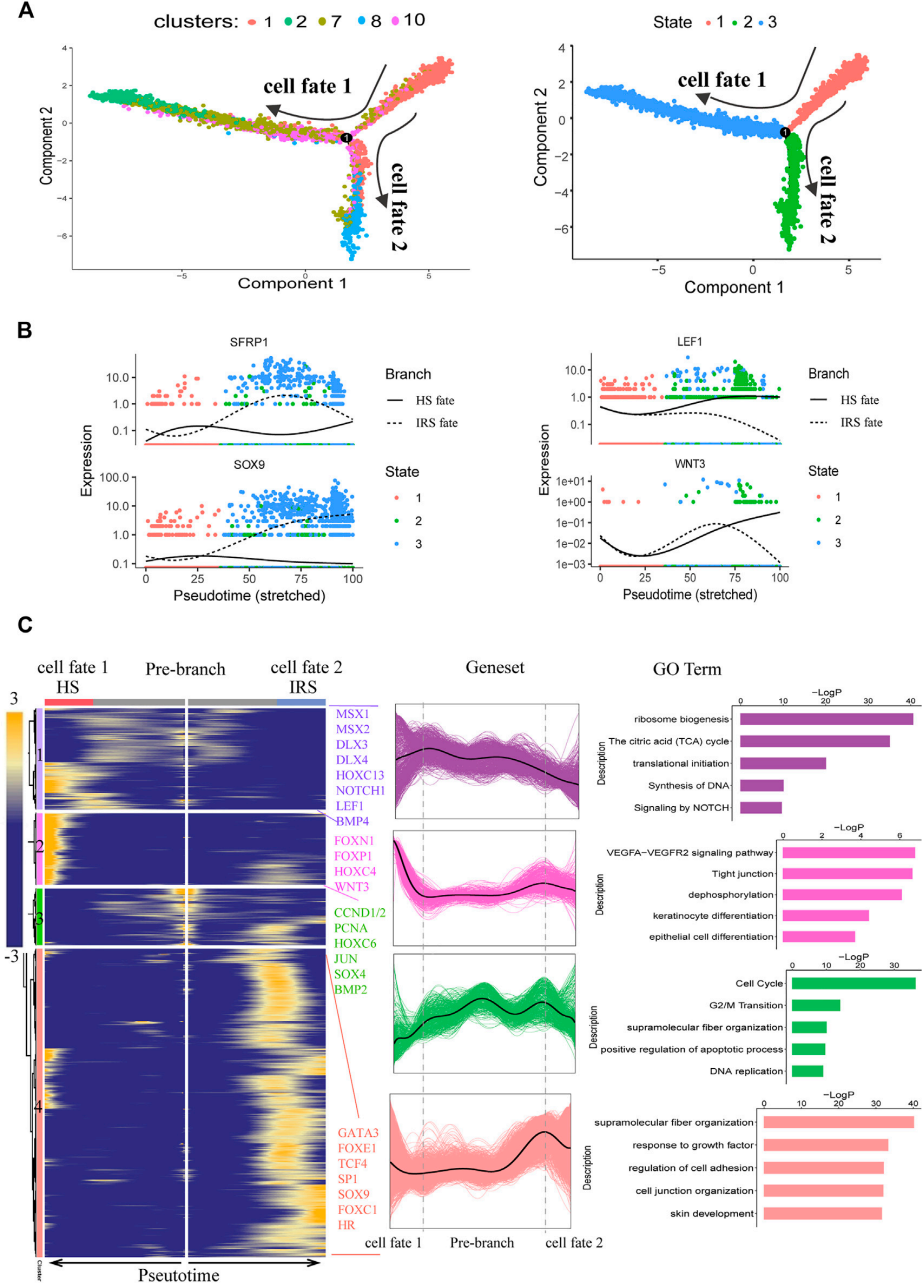
Dissecting IRS and hair shaft fate commitment from matrix precursors. **(A)** Pseudotime visualization of the hair shaft and IRS fate decisions in the pseudotime trajectory. **(B)** The expression profiles of SFRP1, SOX9, LEF1, and WNT3 along pseudotime. Cells are color-coded with cell states, and the solid line represents hair shaft fate, while the dashed line represents IRS fate. **(C)** Heatmap and Go analysis of branch-specific DEGs along pseudotime. Cell fate 1 indicates IRS fate and cell fate 2 indicates hair shaft fate.

After characterizing the different cell clusters, we next sought to identify and characterize the signatures that may govern hair follicle heterogeneity. Since the hair follicle is constantly renewed, once activated, HFSCs typically give rise to matrix progenitors, which then progress to differentiate into their lineages (Avigad Laron et al., 2018). To investigate the gene regulatory machinery underlying the matrix progenitors' commitment to the hair shaft (HS), IRS, and the companion layer, the related hair follicle epidermal cell clusters (clusters 1, 2, 7, 8, and 10) were selected to infer the cell lineage developmental trajectory using a monocle. As expected, pseudotime trajectory construction analysis displayed two branches (Figure 4A). To infer each branch's identity, DEG analysis was performed between the branches (Supplementary Table S2). As a result, IRS marker genes (Moch et al., 2013; Schweizer et al., 2007) including *KRT71*, *KRT25*, *KRT27*, and *GATA3* were enriched in cell fate 1; HS marker genes (Yu et al., 2011) including *KRT36*, *KRT84*, and *KRT2.11* were enriched in cell fate 2, which indicated the successful recapitulation of the HS and IRS differentiation trajectory from the matrix progenitors. Besides, monocle trajectory gene expression analysis revealed HS differentiation-related genes such as *FOXN1*, *HOXC13*, *MSX1*/*2*, and *WNT3* (Hwang et al., 2008; Mesler et al., 2017; Millar et al., 1999; Potter et al., 2011); IRS-differentiation-related genes such as *GATA3* (Kaufman et al., 2003) and unexpected *SOX9*; and matrix cells' enriched genes such as *CCND1*, *CCND2*, and *PCNA* (Supplementary Figure S3). It was of interest that SFRP1, the WNT antagonist (Gopinathan, et al., 2019), was upregulated during IRS specification, while WNT3 and LEF1, the ligand and nuclear mediator of WNT signaling (Nusse, 2012), were upregulated during HS differentiation, which indicated the opposite effect of WNT signaling in matrix progenitors' commitment to the HS and IRS (Figure 4B). To further unmask the molecular mechanism underlying HS and IRS specification, we compartmentalized the identified branch-specific DEGs using k-means clustering (Figure 4C). As expected, gene sets 2, 3, and 4 corresponded with HS, matrix, and IRS, while gene set 1 was speculated relating with the pre-cortex, the precursor cell of HS cortex, since the enriched genes *MSX2*, *HOXC13*, and *DLX3* had been demonstrated as key transcription factors in promoting pre-cortex differentiation (Hwang et al., 2008; Potter et al., 2011; Mesler et al., 2017). To investigate gene functions in each gene cluster, Metascape was used to perform GO analysis. The HS-related gene set (gene set 2) enriched GO terms of “VEGFA-VEGFR2 signaling pathway, Tight junction, keratinocyte differentiation, and epithelial cell differentiation.” The IRS highly expressed genes (gene set 4) had the enriched GO terms “supramolecular fiber organization, regulation of cell adhesion, epithelial cell differentiation, skin development,” and the matrix highly expressed genes (gene set 3) had the enriched GO terms “cell cycle and DNA replication” (Figure 4C). Taken together, our analysis here provided the insights of the gene regulatory machinery underlying matrix cell commitment to HS and IRS in sheep.

### Interaction of the Epidermis and Dermis

**FIGURE 5:**
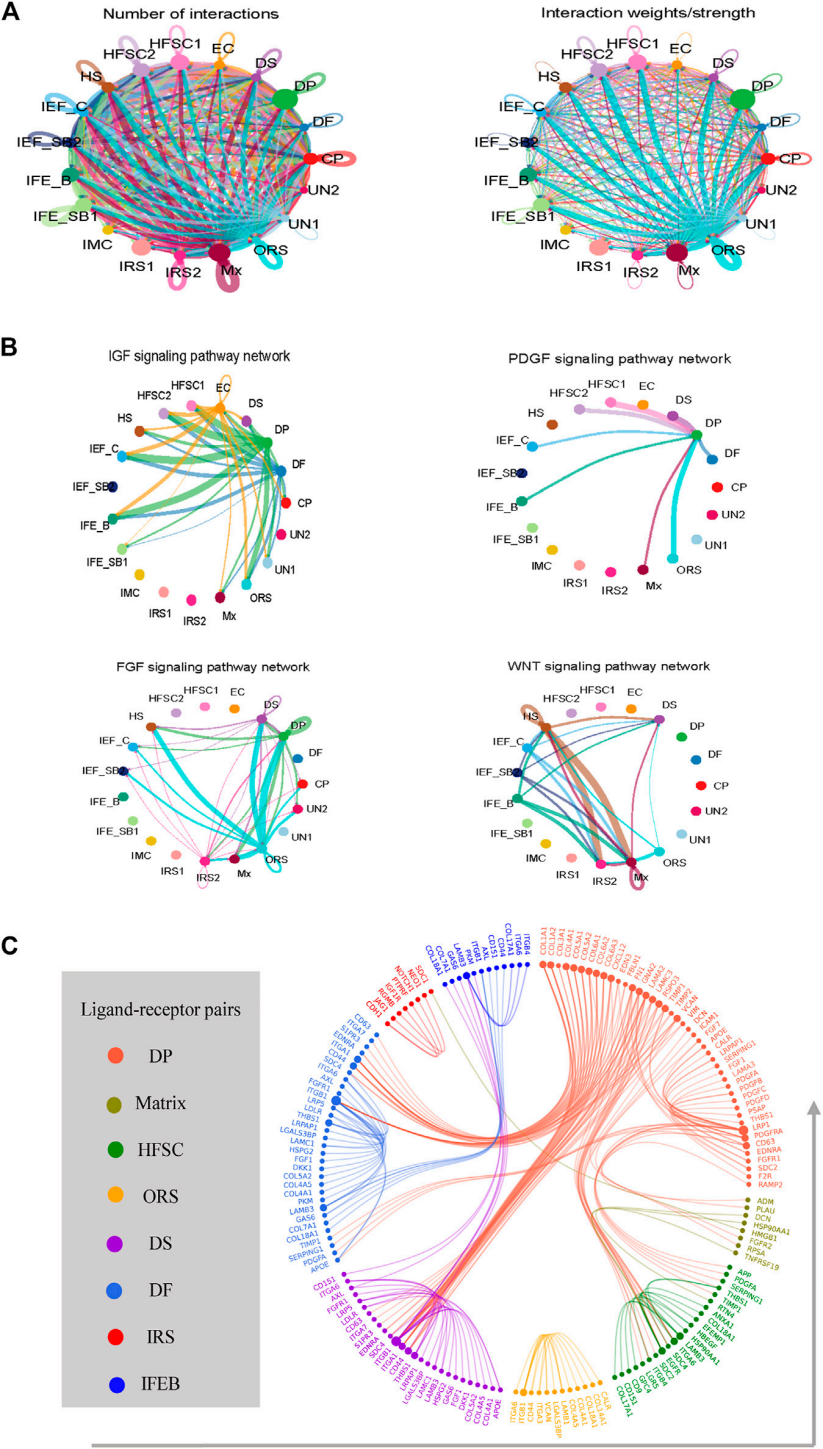
Intercellular ligand–receptor prediction. **(A)** Analyze the number of interactions and interaction strength among different cell populations. **(B)** Identify the signaling pathways among different cell populations. **(C)** Ligand–receptor pairs between main cell populations. Different cell populations are color-coded, and ligand–receptor pairs are linked with a solid line.

Tightly mesenchymal–epithelial interactions orchestrate cyclical hair growth (Arwert et al., 2012; Hsu et al., 2014). To clarify the underlying intercellular communications that drive hair follicle heterogeneity and cell state transitions in sheep skin, we analyzed intercellular communication networks from scRNA-seq data using CellChat. CellChat detected a number of significant ligand–receptor pairs among the 19 cell groups, in which matrix cell and DPC had a clear advantage (Figure 5A). This further emphasized the indispensable roles of DPCs as the signal center. The ligand–receptor pairs were further categorized into 19 signaling pathways, including WNT, IGF, FGF, EGF, PDGF, CXCL, PTN, and VEGF pathways. DP cells may interact with epidermal cells such as HFSC, matrix, and ORS through FGF, IGF, and PDGF signaling, while matrix cells regulate HS cells and IRS cells through WNT signaling (Figure 5B).

To dissect the exact ligand–receptors among different cell types and the cellular source of that ligand, we then used a public ligand–receptor database to infer intercellular communications. By comparing the cell identity specific genes with annotated ligand–receptors, the potential ligand–receptor pairs among different cell populations were sorted (Figure 5C) (Supplementary Table S3). We found strong intercellular communication among dermal and epidermal cell populations including FGF7-FGFR2, and DCN-EGFR as well as collagen family members COL1A1, COL1A2, and COL4A1 and their corresponding receptors. It is worth noting that dermal cells expressed FGF7, which may be a key driver of epidermal differentiation.

### Differentially Expressed Genes in Specific Cells Between Straight Wool and Curly Wool

**FIGURE 6:**
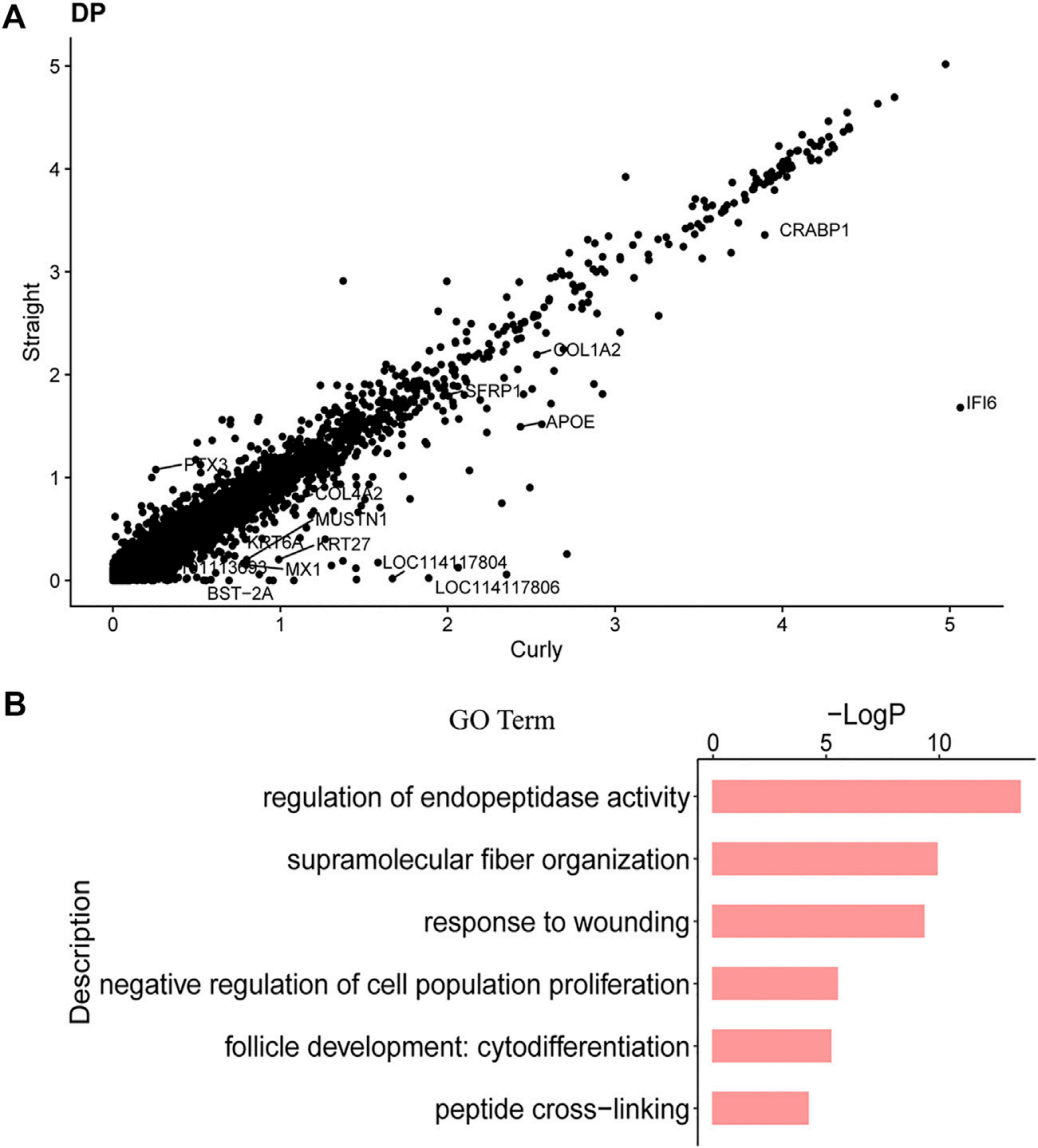
DEGs in DPCs between Straight Wool and Curly Wool. **(A)** Scatter plots show genes that exhibit dramatic differentially expression. **(B)** GO terms for DEGs in DPCs between Straight Wool and Curly Wool.

Previous studies had demonstrated that hair structure and shape in mice had a close relationship with distinct molecular pathways in the DPCs (Driskell et al., 2009). To verify whether the different curvature of the wool was related to distinct molecular pathways in the DPCs, the gene expression profiles of DPCs between straight wool individuals and curly wool were initially compared through Seurat. As a result, 74 DEGs were found (Figure 6A) (Supplementary Table S4). Among these genes, *SPINK5* (Descargues et al., 2005), *RPTN* (Pośpiech et al., 2018), *CRABP1* (Collins and Watt, 2008), *SFN* (Hammond et al., 2012), and *SFRP1* (Bak et al., 2015) had been previously demonstrated to play an important role in hair follicle development. We were particularly intrigued by the *RPTN* gene and *CRABP1*, as variation in the *RPTN* gene may facilitate straight hair formation in Europeans and East Asians (Pośpiech et al., 2018), and *CRABP1* is specifically expressed in the hair follicle DP in normal postnatal skin and may influence regulators of hair bending in mice as retinoic acid-binding proteins (Okano et al., 2012). Meanwhile, a series of collagen genes, including *COL4A2*, *COL3A1*, *COL1A1*, *COL1A2*, *COL6A3*, and *COL4A1*, were found in the DEGs. Collagen, coded by collagen genes, are extracellular matrix molecules that regulate DPCs behavior and hair development (Couchman, 1993). In addition, through comparing the DEGs with DP signature genes, we found that 19 DP signature genes were differentially expressed between straight wool and curly wool, including *S100A4*, *CRABP1*, *SFRP1*, *DCN*, and collagen genes (Supplementary Table S4). To further gain insight into the signature and biological process underlying wool curvature, we used Metascape to perform GO analysis. The DEGs enriched GO terms of “supramolecular fiber organization,” “negative regulation of cell population proliferation,” and “hair follicle development: cytodifferentiation” (Figure 6B), which indicated that the DEGs may govern hair shape through regulating DPCs proliferation and hair follicle differentiation. Furthermore, the DEGs in matrix cell, IRS, and HFSC were also analyzed (Supplementary Tables S5–S9), since these cells may also affect the hair shape. It is worth noting that KRT71, the major structural component of the hair follicle IRS layer, was differentially expressed in the IRS cells between straight wool and curly wool. Since KRT71 had been previously reported associated with the pathological hair structure phenotype and the occurrence of woolly hair (Fujimoto et al., 2012; Harel and Christiano, 2012), the results indicated that DPCs may affect the wool curvature through regulating keratin organization in the IRS. These results will provide a reference for clarifying the molecular mechanism of wool curvature.

## Discussion

For a long time, researchers have been committed to characterize the genes that govern hair follicle development in humans, mice, and sheep, hoping to treat diseases or improve wool production. Meanwhile, the hair follicle is the ideal model for studying systems biology as it undergoes life-long cyclical transformations. Originally, through natural and induced mutant mice as well as the transgenic mice model, a number of genes related with hair follicle development had been identified (Sundberg et al., 2005; Nakamura et al., 2013). Through lineage tracing, microanatomy, and micromechanical separation, the critical cells, DP and HFSC, had been isolated, studied, and characterized *in vitro* (Blanpain et al., 2004; Rompolas et al., 2012; Morgan, 2014; Morita et al., 2021). Recently, Rezza et al. used FACS and RNA sequencing to define gene expression signatures of distinct hair follicle cell types from embryonic skin (Rezza et al., 2016). Rendl et al. examined transcriptional profiles of DPCs and the cells surrounding their niche through employing double-transgenic mice and selective cell-surface labeling (Rendl et al., 2005). However, most of the studies relied on bulk-sampling techniques and cell enrichment using pre-defined markers; the dynamics of genes expression during development and molecular relationship between the different cell populations were still insufficiently addressed. In contrast, recent advances in scRNA-seq technologies have made it possible to comprehensively dissect the cellular composition of complex skin tissues, unveil novel cell populations, and reveal the differentiation and spatial signatures of skin and hair follicle development (Ge et al., 2020; Haensel et al., 2020; Takahashi et al., 2020). However, such studies are lacking so far in sheep; some fundamental questions such as the marker genes of DPC and HFSC, the interaction between epidermis and dermis, and the molecular mechanism of wool curvature on sheep are still unknown.

Here, we used scRNA-seq to sequence 15,830 cells from the lamb skin including straight wool and curly wool skins; 19 clusters were identified, and 14 cell types including all known cell types in hair follicle were subsequently characterized, which showed the cellular heterogeneity of skin cells during tissue homeostasis. Based on Seurat analysis and well-defined markers, we identified the potential marker genes of all the cell types. Among these marker genes, some of these were conserved among different species, such as the keratin markers in different keratinocyte cells (Joost et al., 2020; Takahashi et al., 2020), while some marker genes showed specificity in sheep. Sox2 and CD34 were well-recognized marker genes for DPC and HFSC in mice and humans (Clavel et al., 2012; Sanchez-Danes and Blanpain, 2017), however, we did not detect them in our sequencing data. Besides, through Seurat analysis, we identified two HFSC cell types. Corresponding with that, HFSCs presented quiescent state and proliferate state early in the anagen (Hsu et al., 2011). Recently, Morita traced the origin of HFSC using marker-independent long-term 3D live imaging and scRNA-seq, and upper HFSCs and lower HFSCs with different transcriptome were found (Morita et al., 2021). In addition, our study provided novel marker genes for the different skin cell types in sheep, yet these marker genes should be further verified. Using immunofluorescence of skin tissue sections, several marker genes were verified, which were consistent with scRNA-seq analysis. Furthermore, we successfully applied marker genes identified from our data on DPCs isolated *in vitro*. It is worth noting that IGFBP3, a novel marker gene identified from our data, showed a close relationship with the aggregative growth character of DPCs. In line with that, IGFBP3 was mainly expressed in the human DP (Batch et al., 1996). Previous studies have reported that IGF1 and IGF1 signaling as an important mitogenic and morphogenetic regulator was essential for normal hair growth and development (Su et al., 1999; Weger and Schlake, 2005). Since IGF1 was expressed in the DP from our data and the IGFBP3 have high affinity for IGFs (Wraight, et al., 1994), we speculated that IGFBP3 played a significant role in DPC aggregative growth through IGF signaling.

Based on the above analysis, we identified 19 populations of skin cells and unmasked their gene expression profiles. Nevertheless, the dynamics of genes expression during progenitors' commitment to terminally differentiated keratinocytes were still unknown. Therefore, according to their transcriptional profile, we reconstructed the differentiation processes by ordering hair follicle epidermal cell lineage cells along a path using a network-based approach (Trapnell et al., 2014). We revealed that IRS and HS cells were at the end of branches, while the companion layer was at the front. Correspondingly, Mesler et al. report that matrix can be divided into early and late phases; early matrix progenitors form the hair follicle companion layer, whereas later matrix populations progressively form the IRS and HS (Mesler et al., 2017). Meanwhile, we revealed matrix commitment to the HS and IRS based on distinct temporal, molecular, and functional characteristics. Different transcription factors may govern IRS and HS specification. Signals from the dermal cell were required during epidermal cell lineage commitment (Yang et al., 2017); however, it remains unclear how epidermal–dermal cell interactions drive specific cell fate decisions. Therefore, we analyzed intercellular communication networks using CellChat and the public ligand–receptor database. We revealed that DP cells may interact with the epidermal cell through FGF, IGF, and PDGF signaling, while matrix cells regulated HS cells and IRS cells through WNT signaling. Correspondingly, growth factors and cytokines such as IGF1 and FGF7 secreted from DP regulate the proliferation and differentiation of hair follicle keratinocytes surrounding DP (Schlake, 2005; Weger and Schlake, 2005; Nguyen et al., 2018). In addition, the exact ligand and receptor were inferred, including FGF and IGF pairs.

Besides, we preliminarily explored the molecular mechanism underlying wool curvature. Previously, Demars revealed the mutation underlying fleece variation through comparing ancestral hairy sheep with a long and hairy fleece and modern woolly sheep with a short and woolly fleece in genome-wide scale (Demars et al., 2017). However, the detailed regulatory mechanism is still unclear. Driskell demonstrated that Sox2-positive DPCs specify particular hair follicle type in mice, which indicated that the gene expression profiles may affect hair curvature (Driskell et al., 2009). Due to the selection of lambskin traits, segregation of wool curvature character appeared in the Hu sheep population including curly wool and straight wool individuals, which provide an excellent research material to study the biology of wool curvature. Here, we explored the molecular mechanism underlying wool curvature using curly and straight wool Hu sheep at single cell transcriptome. Different from mice, our study had not detected the specific expression of SOX2 in DPCs. We found 74 DEGs in DPC between straight wool and curly wool, including WNT signaling gene and a series of hair follicle development-related genes. Among these DEGs, Spink5-deficient mice displayed impaired keratinization, hair malformation, and a skin barrier caused by abnormal desmosome cleavage (Descargues et al., 2005). *RPTN*, encoding repetin, could interact with trichohyalin and connect to keratin intermediate filaments, which may facilitate straight hair formation in Europeans and East Asians due to variation in the *RPTN* gene (Pośpiech et al., 2018). *SFN* is a cell cycle regulator involved in the program of epithelial keratinization. Mice heterozygous for the *SFN* mutation had severe defects in HS differentiation (Hammond et al., 2012). *DCN* is expressed in the extracellular matrix of DPCs, where it may play a positive role in hair biology (Zhou et al., 2018). *SFRP1*, the WNT/β-catenin signaling antagonist, is expressed in DPCs, which was related to the capability of inducing hair follicles (Bak et al., 2015). Previous studies showed that WNT/β-catenin signaling maintained the hair-inducing capacity of the DPCs (Kishimoto et al., 2000), was involved in hair follicle differentiation through regulating keratin genes (DasGupta and Fuchs, 1999), and was evidently critical for hair curvature (Nissimov and Das Chaudhuri, 2014; Westgate et al., 2017). In accordance with that, we found that KRT71 was differentially expressed in the IRS cells between straight wool and curly wool groups. Current evidence suggests that KRT71 was involved in the determination of natural variation in hair morphology (Fujimoto et al., 2012; Harel and Christiano, 2012). Based on the above results and the consensus that DPC is the regulatory center of hair follicles, we speculated that different activity of the WNT/β-catenin pathway in the DPCs may affect the keratin organization in the IRS and further affect the wool curvature. Besides, GO analysis was performed on these DEGs. Except for GO terms of “hair follicle development” and “supramolecular fiber organization,” it is worth noting that the DEGs enriched GO terms of “negative regulation of cell population proliferation.” As previous study had demonstrated that the number of DPC specified hair size, shape, and cycling (Chi et al., 2013), we assumed that there is a difference in the number of DPCs between curly wool and straight wool individuals. According to the above analysis, we believe that the DPCs may play a critical role in regulating wool curvature. However, more research is needed for a deeper understanding of wool curvature.

## Conclusion

Taken together, our findings provided an unbiased and systematic view of molecular anatomy of the sheep hair follicle comprising 19 clusters, revealed the differentiation, spatial signatures and intercellular communication underlying sheep hair follicle development, and at the same time revealed the potential molecular mechanism of wool curvature, which will give important new insights into the biology of the sheep hair follicle and its associated breeding.

## Data Availability

The single-cell RNA sequencing data used in this research are deposited in NCBI Gene Expression Omnibus database (https://www.ncbi.nlm.nih.gov/geo/) under accession number: GSE186204. The original contributions presented in the study are included in the article/Supplementary Material; further inquiries can be directed to the corresponding author.
