# Supplementary material for: Single-Cell Transcriptomics Reveals the Molecular Anatomy of Sheep Hair Follicle Heterogeneity and Wool Curvature
Source: Front Cell Dev Biol. 2021 Dec 21;9:800157. doi: 10.3389/fcell.2021.800157 (PMC8724054; doi:10.3389/fcell.2021.800157)
Supplement: Supplementary file 1 [file DataSheet1.zip › Supplementary Figures and Tables/Supplementary figures.pdf]

Supplementary Figure 1

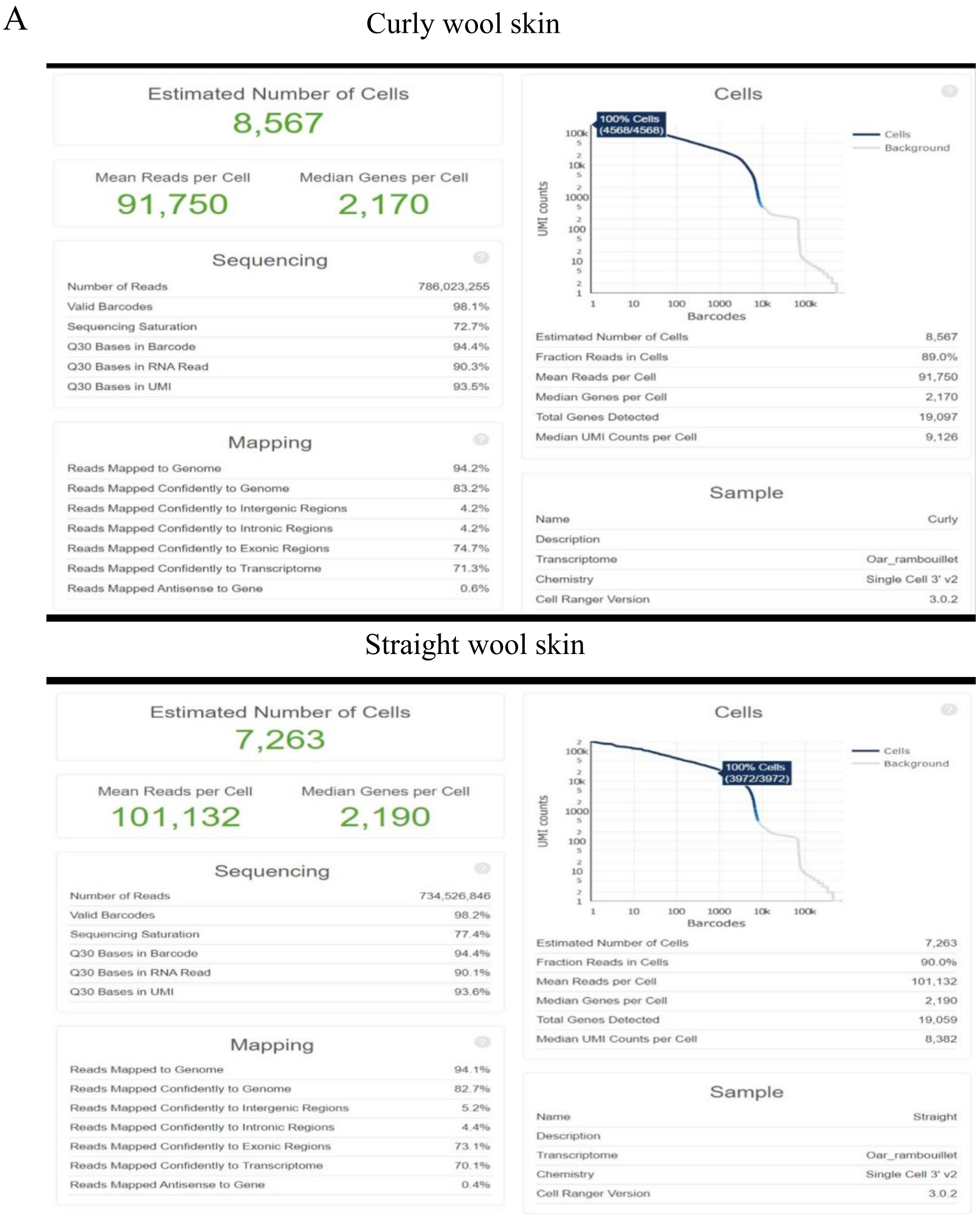


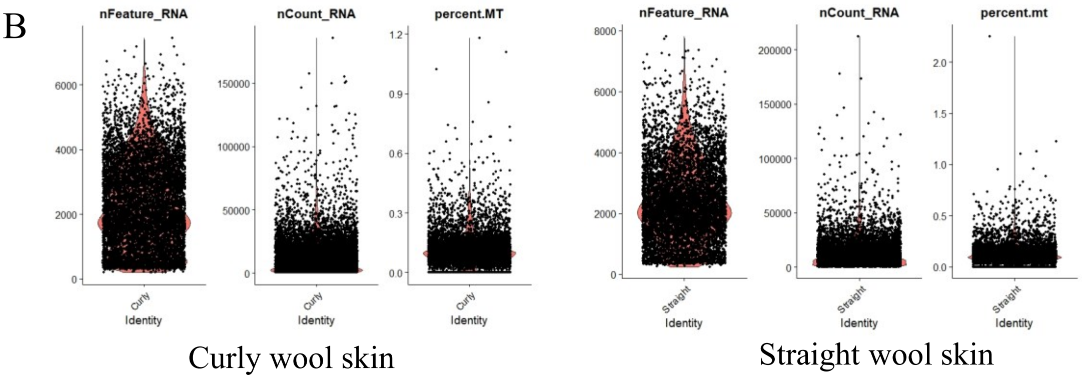


**Supplementary Figure 1** The summary of output of cell Ranger and quality control. (A) The summary of output of cell Ranger. We detected 7,263 single-cell transcriptome profiles for straight wool group and 8567 single cells for waving wool group. (B) Quality control. For each dataset, we filtered cells with unique detected genes less than 200 and genes detected less than 3 cells, total number of detected genes less than 1,750 and percent.mt more than 1.5.

Supplementary Figure 2

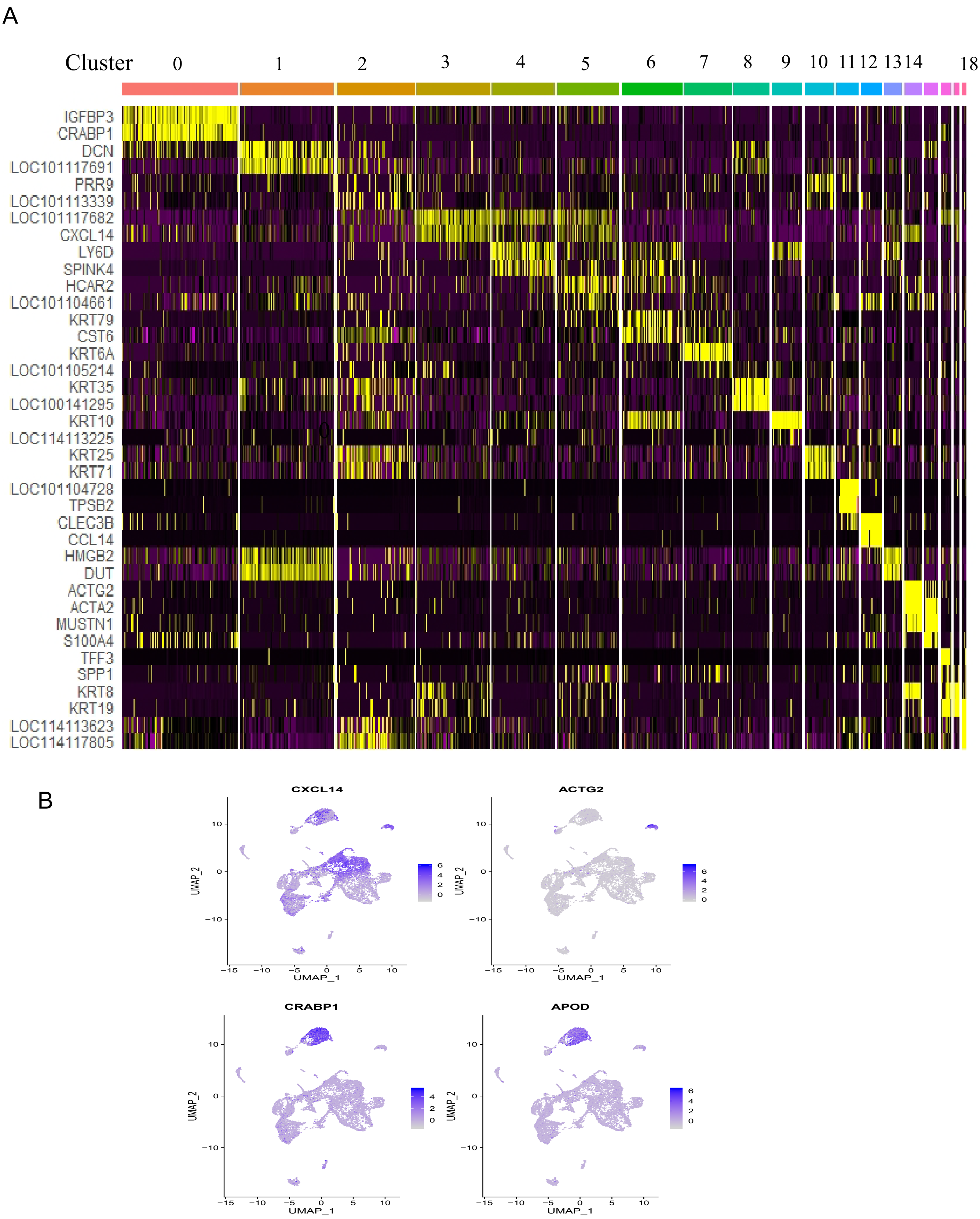


**Supplementary Figure 2** Cell-type identification and gene expression profiles. (A) Clustered heatmap of the top cluster-specific markers of each cluster. (B) UAMP visualization of potential novel markers of cell types.

Supplementary Figure 3

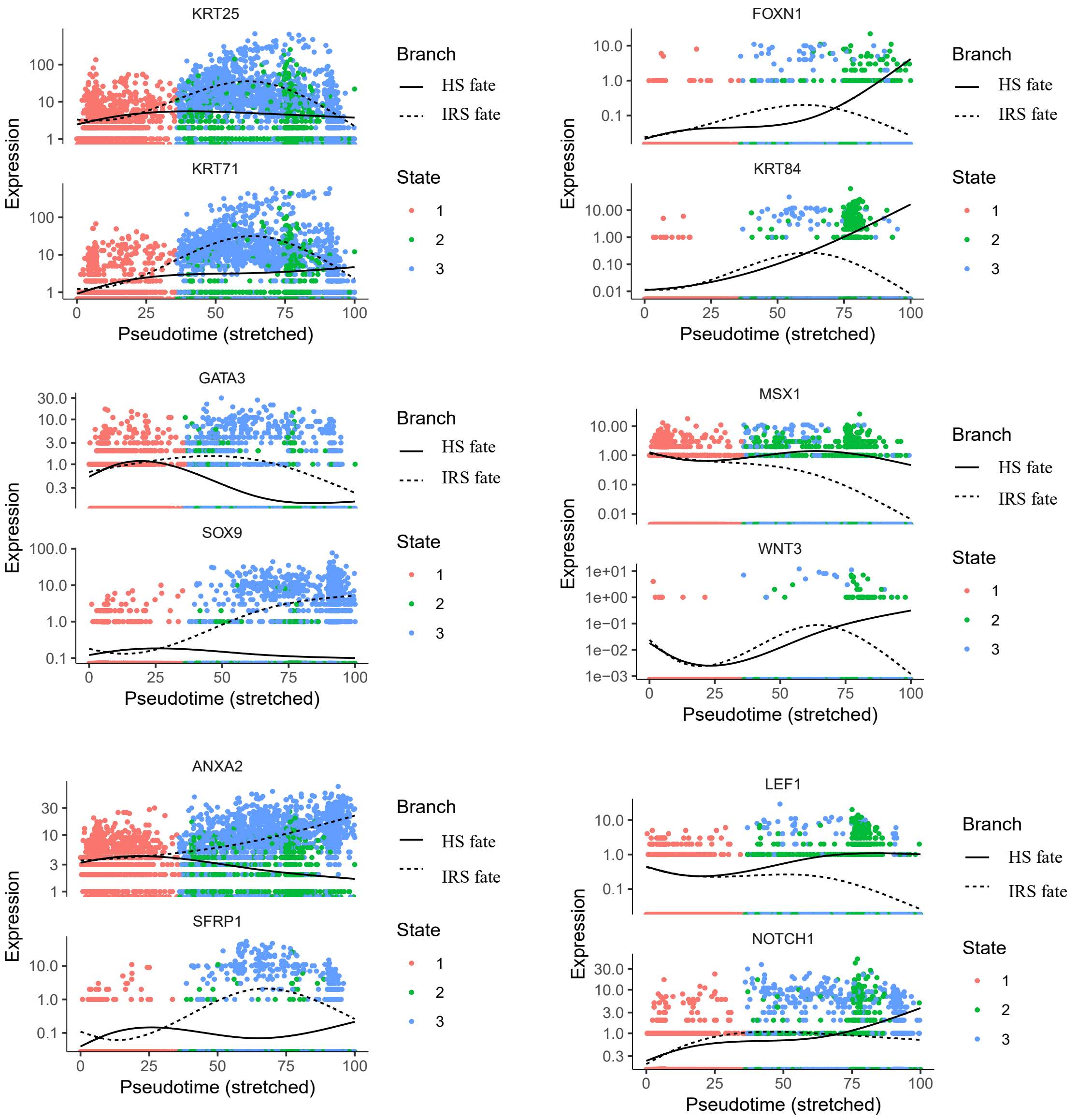


**Supplementary Figure 3** Representative gene expression along pseudotime. Cells were color-coded with cell states and the solid line represents hair shaft fate, while the dashed line represents IRS fate.
